# Archaeognatha of Canada

**DOI:** 10.3897/zookeys.819.23572

**Published:** 2019-01-24

**Authors:** Matthew L. Bowser

**Affiliations:** 1 U.S. Fish & Wildlife Service, Kenai National Wildlife Refuge, Soldotna, Alaska, USA U.S. Fish & Wildlife Service Soldotna United States of America

**Keywords:** Archaeognatha, biodiversity assessment, Biota of Canada, jumping bristletails, Microcoryphia

## Abstract

Current knowledge of the Canadian bristletail (Archaeognatha) fauna is summarized and compared with Tomlin’s 1979 chapter on the group in *Canada and Its Insect Fauna*. Since that time the number of species known from Canada has increased from three to eight. While much work remains to be done to document an estimated eight additional species from Canada, this can be accomplished using an integrated approach.

## Introduction

Substantial progress has been made in our understanding of the bristletail fauna of Canada since Tomlin’s (1979) chapter in *Canada and its insect fauna*, but a great deal of work remains to be done before this fauna is well documented.

Tomlin (1979) reported three species known from Canada and estimated that there were an additional ten species yet to be documented or described. He had considered all Canadian members of the order Microcoryphia (= Archaeognatha) to be in the family Machilidae, but it is unclear whether this was because he differed in opinion regarding the family Meinertellidae proposed by Verhoeff (1910) or because he was not aware of any meinertellid species from Canada at that time.

The one Canadian species mentioned by Tomlin (1979), *Machilisvariabilis* Say, has since been considered unidentifiable because the type material has been lost and Say (1821) did not describe taxonomically useful characters to distinguish this species (see Wygodzinsky and Schmidt 1980). However, it may still be possible to associate this name with an existing species based on the type locality. Say (1821) provided a broad type locality of “probably in almost every temperate part of North America” but specifically included Florida. It is highly probable that this material came from the northeast corner of Florida, where Say had made one collecting trip over the winter of 1817–1818 (Bennet 2002). An unidentified species of *Neomachilellus*, the only archaeognathan besides *M.variabilis* reported from Florida, was later reported from the eastern Florida-Georgia border area (Wygodzinsky 1967, Sturm 1984) and is likely the same species as some of Say’s original types of *M.variabilis*.

North American Archaeognatha are presently a difficult group to work with due to a lack of modern descriptions for some species and inherent challenges of recognizing morphologically similar species. Most progress on the North American fauna since 1979 has been due to the work of Pedro Wygodzinsky and Helmut Sturm, both experts on this group working at a worldwide scope. Wygodzinsky and Schmidt (1980) published the only modern regional treatment applicable to Canadian bristletails, covering the northeastern United States and adjacent provinces of Canada. More recent work by Sturm and others pertaining to the Canadian fauna (Sturm 1991, 2001, Sturm and Bach de Roca 1992, Sturm and Bowser 2004) has been incremental, with additions of species and treatment of one genus (*Mesomachilis* Silvestri).

A total of eight species of bristletails are now known from Canada, representing two families (Table 1). Of these, two species were introduced from the Palearctic to the east coast of North America, apparently in ship ballast material (Wygodzinsky and Schmidt 1980). No species in the Canadian fauna are known to be widespread across Canada; most appear to be restricted to defined ecological zones. Distinct bristletail assemblages are present in the Pacific Maritime, Western Interior Basin, and Montane Cordillera ecozones.

**Table 1.** Census of Archaeognatha in Canada.

Taxon^1^	No. species reported in Tomlin (1979)	No. species currently known from Canada	No. BINs^2^ available for Canadian species	Est. no. undescribed or unrecorded species in Canada	General distribution by ecozone^3^	Information sources^4^
Machilidae	3	7 (2)	9	7	southern Canada from Pacific Maritime to Atlantic Maritime	Wygodzinsky and Schmidt 1980, Sturm 1991, Sturm 2001, Chlebak 2013; specimens in DEBU, RBCM, UBCZ
Meinertellidae	0	1	1	1	Pacific Maritime, Western Interior Basin, Prairies	Sturm and Bach de Roca 1992, Acorn 2011; specimens in DEBU, RBCM; observations on iNaturalist.org
**Total**	**3**	**8 (2)**	**10**	**8**		

**^1^**Classification follows Sturm and Machida (2001). **^2^**Barcode Index Number, as defined in Ratnasingham and Hebert (2013). ^3^See figure 1 in Langor (2019) for a map of ecozones. **^4^**Data source collection codens: DEBU, University of Guelph; RBCM, Royal BC Museum; UBCZ, University of British Columbia, Spencer Museum.

There are few DNA barcodes for Canadian bristletails. Ten BINs (Barcode Index Numbers) of bristletails have been obtained from Canada, only two of which have been associated with accepted species names. Some of the unidentified BINs will likely be eventually identified as previously described species, but some likely represent undescribed species. DNA barcode sequences from the two Palearctic species established in eastern Canada have been obtained from elsewhere but not yet from Canada.

The author is aware of six potentially undescribed species: two entities in the genus *Petridiobius* Paclt represented by the BINs BOLD:AAV1529 and BOLD:AAV1531 from the Canadian Rockies; specimens representing one of two BINs BOLD:AAV1528 and BOLD:ACJ4257 from coastal British Columbia (BC) that are indistinguishable from the original description of *Pedetontussubmutans* Silvestri; a *Mesomachilis* sp. and a species of *Pedetontoides* Mendes from the Western Interior Basin ecozone of British Columbia (BC); and a species similar to *Leptomachilis* Sturm from Kootenay National Park represented by BIN BOLD:AAV1530. More species are likely to be found in Canada, especially in regions with complex glacial history, a situation that has led to high species diversity of bristletails in the European Alps (Wachter et al. 2012, Gassner et al. 2014, Dejaco et al. 2016).

Dejaco et al. (2012, 2016) and Gassner et al. (2014) have recently demonstrated success in discriminating among morphologically similar species of bristletails using an integrated approach incorporating multiple morphometric and molecular methods. Appropriate next steps toward improving our understanding of the Canadian archaeognathan fauna would be to collect high-quality specimens that are suitable for both morphological and molecular methods, then apply an integrated taxonomic approach to produce treatments which include identification keys. Areas where additional collecting would be most helpful include the Western Interior Basin and Montane Cordillera ecozones, apparently home to the greatest diversity of Canadian bristletails; the Prairies ecozone, where bristletails are known (Acorn 2011) but have neither been DNA barcoded nor identified to species; and the Atlantic Maritime ecozone, from which no bristletail specimens have been DNA barcoded. While Tomlin’s (1979) concluding remark regarding the Canadian bristletails that, “obviously much work remains to be done in this group”, remains true today, fortunately tools are now available to complete this work much more satisfactorily.
